# Interspecific competitive interactions affect body size and oxidative status of two nonnative salmonid species

**DOI:** 10.1007/s10695-024-01301-0

**Published:** 2024-01-19

**Authors:** Marco Parolini, Rocco Iacobuzio, Bruno Bassano, Roberta Pennati

**Affiliations:** 1 Department of Environmental Science and Policy, University of Milan, Via Celoria 2, 20133 Milan, Italy; 2 Parco Nazionale Gran Paradiso, Via Pio VII 9, 10135 Turin, Italy

**Keywords:** Allopatry, Antioxidant defenses, Brown trout, Sympatry, Rainbow trout

## Abstract

In fish, interspecific interactions between nonnative and other sympatric species are considered determinants in shaping species assemblages. Such interactions can also arise between nonnative fish species only, including salmonids such as the brown trout (*Salmo trutta*, Linnaeus, 1758) and the rainbow trout (*Oncorhynchus mykiss*, Walbaum, 1792), returning contrasting outcomes. The present manipulative experiment was aimed at exploring the effect of interspecific competition on the body growth and the oxidative status of parr (2 + -year-old individuals) of the brown trout and the rainbow trout. Allopatric (intraspecific competition) and sympatric (interspecific competition) populations of these species were experimentally recreated in two wild streams. At the end of a 2-month-long experiment, changes in specific growth rate (SGR), oxidative status (i.e., levels of reactive oxygen species and activity of antioxidant enzymes such as superoxide dismutase — SOD, catalase — CAT and glutathione peroxidase — GPx) and oxidative damage (i.e., lipid peroxidation) were investigated in brown and rainbow trout individuals maintained in allopatric or sympatric populations. Sympatric interactions between rainbow and brown trout parr resulted in a significant decrease in SGR of brown trout individuals only. Moreover, an overall modulation of the oxidative status, in terms of an increase in ROS levels coupled with the activation of SOD and CAT activity, occurred in brown trout individuals under sympatric conditions. These findings might suggest that, under sympatric conditions, parr of the rainbow trout are more competitive than brown trout for food acquisition. However, this competition affected the antioxidant defenses of the brown trout only, probably because of reduced ingestion of dietary antioxidants or increased physical activity and aggressive behavior. Thus, interspecific interactions can induce physiological and phenotypic effects on parr of nonnative salmonids, with potential consequences on the establishment of populations of these species in freshwater ecosystems.

**Supplementary Information:**

The online version contains supplementary material available at 10.1007/s10695-024-01301-0.

## Introduction

Interspecific interactions between nonnative and other sympatric species are considered determinants of the successful establishment of nonnative species, as well as of the replacement of native and/or nonnative species by other nonnative species introduced later (Shea and Chesson 2002). A number of studies focused on the effects of interspecific interactions between nonnative and native species (e.g., Gurevitch and Padilla 2004), and some of them focused on salmonids. Different nonnative salmonid species were introduced into nonindigenous areas as fisheries resources, where they coexist and interact with native salmonid species (Korsu et al. 2008). The brown trout (*Salmo trutta*, Linnaeus, 1758) and the rainbow trout (*Oncorhynchus mykiss*, Walbaum, 1792) are two of the most common nonnative species introduced in freshwater ecosystems worldwide so they were both listed as 100 of the World’s Worst Invasive Alien Species by the International Union for Conservation of Nature (IUCN) (Lowe et al. 2000). Because of their invasiveness (Hasegawa 2020), some studies explored negative consequences due to interspecific interactions between these two nonnative species and some native species of salmonids. For instance, their introduction negatively affected the populations of the white-spotted charr (*Salvelinus leucomaenis*) and the masu salmon (*Oncorhynchus masou*) (Miyasaka et al. 2003; Inoue et al. 2009, 2022; Morita et al. 2016; Hasegawa and Maekawa 2008), as well as the population of the grayling (*Thymallus thymallus*) and landlocked Atlantic salmon (*Salmo salar*) (Hagelin and Bergman 2021). Competitive interactions can also occur with different intensities between these two nonnative species, with different outcomes depending on their life stages. At early life stages, the interactions between these species result in a replacement of the rainbow trout by the brown trout because of the shift towards unsuitable habitats (Fausch 2007) and hampered recruitment (Gatz et al. 1987) of the rainbow trout. At the adult stage, the rainbow trout overcomes the brown trout through redd superimposition (Scott and Irvine 2000) and predation of fry (Avila et al. 2018). Moreover, a manipulative study by Hasegawa (2016) demonstrated that competitive interactions between young-of-the-year (YOY) of these species resulted in a decrease of rainbow trout stomach content in coexistence (i.e., sympatry) with the brown trout, while decreased growth rate occurred in high-density conditions in allopatry and sympatry with the brown trout. In contrast, the stomach content and the growth rate of the brown trout were not affected neither by the interaction with the rainbow trout nor by total fish density (Hasegawa 2016). These results suggest that, under sympatric conditions, the brown trout is more competitive for limited food resources, forcing the rainbow trout to be replaced or excluded from the habitat (McDowall 2003).

At the individual level, the competitive interactions for limited resources result in physiological challenges that can affect energy allocation to key biological processes, such as the growth and the development (Kassahn et al. 2009), triggering a generalized stress response that leads the individual to intake the energy necessary for vital functions through the mobilization of glucose (Kassahn et al. 2009). However, repeated or chronic stress can impose a high-energy demand to the individual (Barton 2002; Barton et al. 2002; Cook et al. 2012). Thus under conditions of food limitation, energy reserves cannot be sufficient to maintain the homeostasis (DiBattista et al. 2006), with potential negative consequences on growth, physiology, and timing of sexual maturation (Barton 2002; McGhee and Travis 2011). Competitive interactions for food can also result in an oxidative stress condition, which is defined as the imbalance between reactive oxygen species (ROS) and antioxidant defenses in favor of the former (Chowdhury and Saikia 2020). In particular, according to the theory of “dietary oxidative stress,” the oxidative status of the organisms can be altered because of an insufficient supply of nutrients (Sies et al. 2005). Many antioxidants contributing to the total antioxidant capacity of fish originate from the diet, especially fat-soluble antioxidants that cannot be synthesized de novo by animals (Goodwin, 1984). Therefore, modulations of food availability and/or food quality can result in changes in acquiring or producing antioxidants, exposing the organism to a wide range of physiological impacts related to oxidative stress, most of which are attributable to the overproduction of ROS and the inability to counteract their toxicity (Robinson et al. 1997). Moreover, competitive interactions can increase physical efforts related to crucial issues of fitness and survival of fish, including foraging activity, predator avoidance, or reproduction, leading to an oxidative stress condition due to enhanced oxygen metabolism at the level of the mitochondria (Alessio and Goldfarb 1988).

The present manipulative experiment was designed to examine if competitive interactions can affect the phenotype and induce oxidative stress in parr (2 + years old individuals) of two nonnative salmonid species, i.e., the brown trout (*Salmo trutta*, Linnaeus, 1758) and the rainbow trout (*Oncorhynchus mykiss*, Walbaum, 1792). Allopatric and sympatric populations of these trout species were recreated in pools into two small streams flowing in the territory of the Gran Paradiso National Park (Northwestern Italy). The low density of individuals under allopatric and sympatric conditions was maintained in all the experimental pools (< 1 individual/m^2^) for about 2 months. Although density-dependent effects of competition have been demonstrated in salmonids (e.g., Grant and Imre 2005; Hasegawa 2016; Grossman and Simon 2020), the outcomes obtained at high density represent a consequence of the inherent tradeoffs of experimental ecology (Underwood 2012), and they are not representative of the interactions occurring under natural conditions (McHugh and Budy 2006). Thus, in order to return ecologically relevant results and to enlarge the knowledge on the outcomes of interspecific competition between two nonnative salmonid species, we preferred to recreate low-density populations of trout. At the end of the experiment, trout were sampled to assess potential differences in specific growth rate (SGR) and oxidative stress-related endpoints (i.e., hepatic and gill levels of reactive oxygen species — ROS, activity of the antioxidant enzymes superoxide dismutase — SOD, catalase — CAT and glutathione peroxidase — GPx, and lipid peroxidation) between individuals of both the species maintained in allopatry or sympatry. Although a previous study demonstrated that intra- and interspecific competition could affect the food ingestion and the growth rate of YOY individuals of the rainbow trout, but not of the brown trout, we have no a priori expectations on the outcomes of interactions on the body growth of parr. Similarly, we have no a priori expectations on the rise of oxidative stress in trout maintained in allopatry or sympatry because of the lack of information concerning this condition in fish experiencing competitive interactions.

## Materials and methods

### Experimental design

**Fig. 1:**
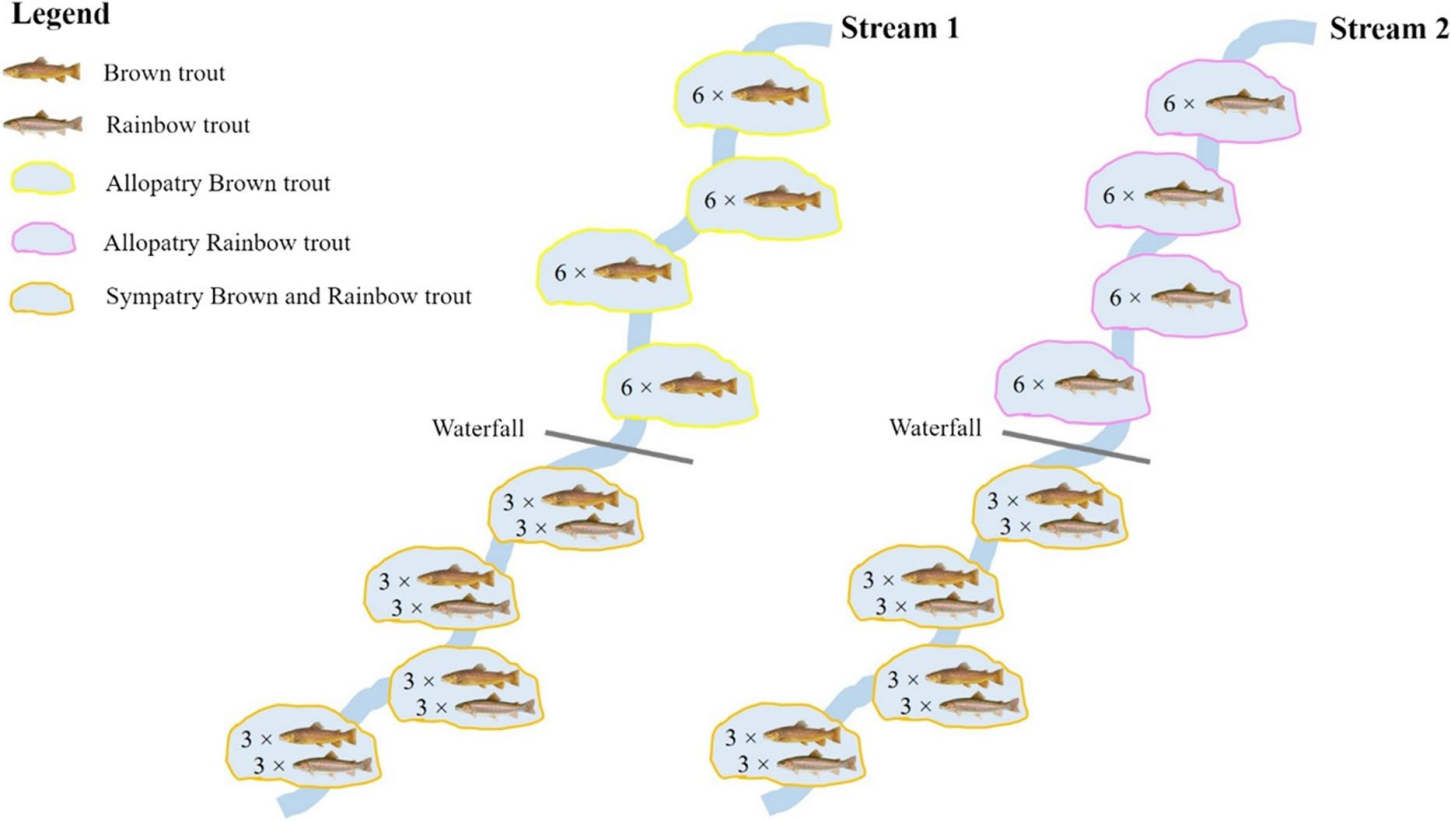
Schematic representation of the experimental plan to assess the effects of competition between two nonnative trout species

The present experiment was performed between September and November 2016 within the territory of the Gran Paradiso National Park (Northwestern Italy), specifically in the Orco River valley. To investigate the effects of competition between the brown and the rainbow trout, the relationships occurring between the individuals of the species were experimentally manipulated in two small streams, recreating allopatric and sympatric conditions. In allopatric conditions, populations exclusively composed of brown or rainbow trout individuals were created in different pools, while in sympatric conditions, brown and rainbow trout individuals coexisted in the same pool. The experiment relied on a mark–recapture sampling approach. First, two experimental streams (Fig. 1) were identified, namely the Rio Combetta (hereafter identified as stream 1 or S1; latitude 45° 27′ 34.64″ N; longitude 7° 9′ 50.04″ E) and Rio della Percia (hereafter identified as stream 2 or S2; latitude 45° 27′ 35.79″ N; longitude 7° 9′ 59.61″ E). Both the streams had similar hydrological characteristics in terms of flow, substrate, water regime, and water chemical-physical features, and they were characterized by the typical riffle-pool morphology of mountain streams. The presence of the focal species and other salmonids (i.e., the brook trout *Salvelinus fontinalis*) in S1 and S2 was preliminarily checked through electrofishing activity. Each stream was divided into transects of about 100 m in length that were travelled thrice at 30-min intervals to collect all the salmonids that formerly populated S1 and S2. All the salmonids collected in S1 and S2 were removed and transferred downstream of the experimental pools. Then, to re-create salmonid populations in S1 and S2, individuals of brown and rainbow trout were sampled using a backpack electrofishing unit in the upper part of the Orco River (latitude 45° 27′ 32.4″ N; longitude 7° 9′ 18″ E) flowing in the territory of the Gran Paradiso National Park, where the species coexist. Trout used in our experiment did not come from hatchery but they came from resident populations. As these populations were not supplemented by hatchery fish and were not affected by fishery activity for a long time (Parolini et al. 2018a,b; 2019), we had a unique opportunity to study the effects of interspecific competition between two salmonid species living under a natural selection regime. As a preliminary study of the population structure in this sampling site showed that the most abundant age class for both the species was parr (i.e., ca. 20% for the brown trout and ca. the 50% for the rainbow trout; Iacobuzio 2017), we sampled only individuals belonging to this specific age class to be used in the experiment. After sampling, trout were transferred to a 100 L tank equipped with an oxygenator and transported nearby the experimental streams within 15 min. Then, the trout were anesthetized dissolving clove oil in a water bucket (0.05% v/v) for body size measurements and individual marking. The body size, in terms of total weight and total length, was measured for all the individuals selected for the manipulative experiment. Then, each single trout was marked with colored elastomers (Scubla Srl, Italy), which were injected under the epidermis of the gill operculum or close to the first ray of the pectoral fin. Different combinations of colors (i.e., yellow, blue, pink, or red) and position of the mark (i.e., right or left operculum/pectoral fin or cross combinations) were randomly assigned to each trout for unequivocal identification. After marking, the trout were placed in pools identified into the experimental streams.

To test the effects of interspecific competitive interactions between the rainbow and the brown trout, we had to re-create three different experimental conditions: (1) brown trout in allopatry, (2) rainbow trout in allopatry, and (3) rainbow and brown trout in sympatry. As in the study area, we did not find three streams to assign a single treatment each, we designed a “within-stream design” to test for the effects of allopatric and sympatric conditions. This approach allowed us to test for the effects of interactions limiting the effect of the location because trout in allopatric and sympatric conditions experienced the same environment.

Eight pools with similar morphometric features (i.e., size and water depth) and suitable for hosting trout at selected densities were preliminarily identified in S1 and S2. Diverse, putatively suitable pools to host the trout populations identified but we selected those that were at least 20 m far from each other to preclude direct interactions between trout in consecutive and/or close pools (e.g., some trout could move from the pool of origin to another one altering the competition intensity within the pools). Selected pools can be considered independent. No individual was collected in a pool different from the original one where it was stocked.

The average depth and area of the pools designed to host sympatric populations were similar and they were 43.2 ± 9.1 cm and 17.5 ± 8.7 m^2^, respectively, while for pools hosting allopatric populations, they were 42.1 ± 11.1 cm and 13.2 ± 9.3 m^2^, respectively. Both the streams were divided into two sections that were isolated by a waterfall precluding the migration of the trout, at least upstream. Four pools were identified downstream of the waterfall, whereby sympatric conditions were recreated, while in the four pools identified upstream of the waterfall, allopatric conditions were recreated (Fig. 1). No individual included in the allopatric pools was found in sympatric ones and vice versa.

Specifically, in S1, allopatric conditions for brown trout were planned, while in S2, the same conditions were created for the rainbow trout. As the population density within the pool can affect the outcomes of interspecific competition between the brown trout and rainbow trout, at least in YOY individuals (Hasegawa 2016), a low density of individuals was maintained in experimental pools (< 1 individual/m^2^). Thus, similar trout densities were created for sympatric (0.4 ± 0.2 individuals/m^2^) and allopatric conditions (0.5 ± 0.3 individuals/m^2^). Six individuals of the same species were stocked into the pools designed to support an allopatric population of brown trout (allopatry brown trout) or rainbow trout (allopatry rainbow trout). To achieve a similar total trout density (i.e., across treatments within streams) and an approximately equal ratio of brown to rainbow trout within sympatric pools (see McHugh and Budy 2006), three individuals of both the species were stocked in pools supporting sympatric populations (sympatry brown and rainbow trout). In total, 48 fish were introduced into each experimental stream. A rock dam placed at the outlet of the pool was built to preclude the escape of the trout from the pools. As the body size of the trout can affect social interactions, we introduced in the experimental pools individuals of the same age class and body size (see *Results* section). The experiment started on September the 7th (in S1) and the 13th (in S2), and ended on November the 2nd (both S1 and S2). Specifically, in S1, the experiment lasted 56 days, while in the S2, only for 50 days. These differences depended on adverse weather conditions that precluded sampling and setting up the experiment simultaneously in the two streams and constrained to conclude the experiment in S2 a week earlier than in S1. At the end of the experiment, trout were recaptured by electrofishing activity, anesthetized with clove oil and, after body size measuring, they were euthanized. The specific growth rate (SGR) was calculated for all the recaptured individuals using the formula reported by Hasegawa (2016):

$$\mathrm{SGR}\;=\;100\;\left(\ln\;M_1-\;\ln\;M_0\right)/\mathrm t$$

where *M*_0_ and *M*_1_ are body mass at the beginning and at the end of the experiment, respectively, and *t* is the number of days the fish were maintained in the experimental pools. Fish were transferred in a laboratory and dissected. The liver and the gills were isolated, quickly frozen in liquid nitrogen, and maintained at − 80 °C until a biochemical analysis of oxidative stress was performed.

### Oxidative stress biomarkers in the liver and gills of trout

The levels of reactive oxygen species (ROS), the activity of the main antioxidant enzymes (SOD, CAT, and GPx), as well as the levels of lipid peroxidation, were measured in triplicate in homogenates of gills and liver dissected by rainbow trout and brown trout individuals according to the methods described by Parolini et al. (2018a; 2019). Gills and liver (≈1 g fresh weight) were homogenized in 100 mM phosphate buffer (pH 7.4) with the addition of 100 mM KCl and 1 mM EDTA, protease inhibitor cocktail (1:10 v/v), and dithiothreitol (DTT, 100 mM). Homogenates were centrifuged at 45,000 × *g* for 1 h at 4 °C. The supernatant was then collected to assess the protein content according to the Bradford method (1976), using bovine serum albumin (BSA) as a standard. The levels of reactive oxygen species (ROS) were assessed through the dichlorofluorescein-diacetate (DCFH-DA) method according to Deng and co-authors (Deng et al. 2009). An aliquot of the crude homogenate was centrifuged for 20 min at 15,000 × g at 4 °C. Then, 20 µL of the homogenate was added to a 96-well plate and incubated for 5 min at room temperature before adding 100 mL of PBS and 8.3 mL of DCFH-DA (10 mg/mL in DMSO). The plate was incubated at 37 °C for 30 min; the fluorescence intensity was measured by an Infinite® 200 PRO microplate reader (TECAN Life Sciences) at excitation *λ* = 485 and emission *λ* = 536 nm wavelength. The ROS concentration was expressed in arbitrary units as AU DCF/mg proteins. Enzyme activities and lipid peroxidation were assessed through spectrophotometric methods (Parolini et al. 2019). Briefly, the inhibition of the cytochrome c (10 µM) reduction caused by the superoxide anion generated by the xanthine oxidase (1.87 mU/mL)/hypoxanthine (50 µM) reaction at *λ* = 550 nm was used to measure the activity of SOD. The SOD activity was expressed as SOD units/mg protein, whereby a SOD unit corresponds to the 50% inhibition of the xanthine oxidase/hypoxanthine reaction. The CAT activity was measured by monitoring the consumption of hydrogen peroxide (50 mM) at *λ* = 240 nm. The CAT activity was expressed as µM/min/mg protein. The GPx activity was assessed by monitoring the consumption of NADPH at *λ* = 340 nm, using hydrogen peroxide (0.2 mM) as a substrate in phosphate buffer (50 mM; pH 7) added with glutathione (2 mM), sodium azide (1 mM), glutathione reductase (2 U/mL), and NADPH (120 µM). The GPx activity was expressed in µM/min/mg protein. Levels of lipid peroxidation were measured through the thiobarbituric acid-reactive substances (TBARS) on raw homogenates of both the trout organs. The absorbance of the solution was read at *λ* = 535, and lipid peroxidation was expressed as nmol TBARS formed/g fresh weight.

### Statistical analysis

The effects of interspecific competition between brown trout and rainbow trout individuals on body growth (i.e., specific growth rate or SGR) and oxidative stress-related endpoints (i.e., levels of ROS and lipid peroxidation, the activity of SOD, CAT, and GPx) were tested through the application of generalized linear models (GLMs). As the brown and the rainbow trout can experience different growth rates and oxidative stress, GLMs were run separately for each species. The experimental condition (i.e., allopatric or sympatric condition) was included in the models as a fixed effect factor. The sex of the fish was not included in the models because we had no a priori expectation of sex-related differences in the response to competition. Moreover, most of the trout of both the species we used in the experiments had immature gonads, precluding sex identification. In models assessing the effects of interspecific competition on SGR, we included the Fulton’s condition factor (K-factor, based on length–weight relationships; Bolger and Connolly 1989) at the beginning of the experiment as a covariate to take into account the initial body size of the individuals. The same models were also run including the initial weight of the trout as a covariate, returning qualitatively similar results (data not shown). As the duration of the treatment was slightly different for the two streams (i.e., stream 1 — S1 and stream 2 — S2) we used for the manipulative experiment (the experiment lasted 56 days in the S1 and 50 days in the S2), in all the statistical models we preliminarily included the duration of the experiment as a factor. Similarly, we also preliminarily included the population density in the different experimental pools as a covariate. As no significant effect of the duration of the experiment and population density was noted, we removed both from the final models in a single step. The final GLMs on SGR included the experimental condition as a predictor and the K-factor as a covariate, while the final GLMs on biochemical analyses performed on the liver or the gills included the experimental condition as a predictor only. Statistical analyses were run in R 3.6.1 (R Core Team, 2019) using the *lmer* package.

## Results

At the beginning of the experiment, the mean body mass (± standard deviation; to the nearest 1 g) of the rainbow trout in pools recreating allopatric conditions (57 ± 26 g) did not significantly differ compared to that of conspecific in pools recreating sympatric conditions (66 ± 23 g; paired *t*-test; *t* = 1.663, *p* = 0.100; considering individuals from both the streams). Similarly, the mean body mass (± standard deviation) of the brown trout in pools recreating allopatric condition (56 ± 18 g) did not significantly differ compared to that of conspecific in pools recreating sympatric condition (65 ± 21 g; paired *t*-test; *t* = 1.550, *p* = 0.185). In addition, no significant differences in body weight occurred between rainbow and brown trout placed in allopatric (paired *t*-test; *t* = 0.097, *p* = 0.992) and sympatric pools (paired *t*-test; *t* = 0.186, *p* = 0.852). Similar results were obtained for the total length of the trout. The mean length (± standard deviation; to the nearest 1 mm) of rainbow trout in allopatric and sympatric conditions was 17.1 ± 2.8 cm and 17.5 ± 3.0 cm, respectively, while for the brown trout was 17.6 ± 3.0 cm and 18.8 ± 2.5 cm, respectively. No significant differences in the total length among individuals maintained under different experimental conditions were noted (paired *t*-test; *t* < 1.887, *p* > 0.063 for all the pairwise comparisons).

**Fig. 2:**
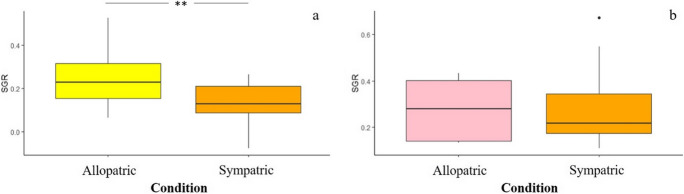
Boxplots of the effects due to interspecific competition on the specific growth rate (SGR) of the brown (**a**) and rainbow (**b**) trout. The asterisk indicates a statistically significant difference (**p = 0.010) between individuals of the two trout species under allopatric or sympatric conditions

Overall, at the end of the experiment, the 70% of trout we stocked in the pools of the streams (= 67/96) were recaptured. Specifically, the 73% in allopatric conditions (= 35/48), and the 67% in sympatric conditions (= 32/48). We can speculate that not-recaptured individuals could be hidden deep in the hole, precluding their capture through electrofishing, but we could not exclude that some of them were dead. A significant effect of experimental condition (i.e., allopatric *vs* sympatric; *F*_1,28_ = 7.633, *p* = 0.010) on SGR of the brown trout was noted, with individuals experiencing sympatric condition that grew less than conspecifics under allopatric ones (Fig. 2a). In contrast, interspecific competition did not affect the SGR of rainbow trout individuals (*F*_1,24_ = 0.011, *p* = 0.916; Fig. 2b). The initial body size of trout, in terms of K-factor, did not predict the SGR (*F* < 2.762; *p* > 0.110 for both the species).

**Fig. 3:**
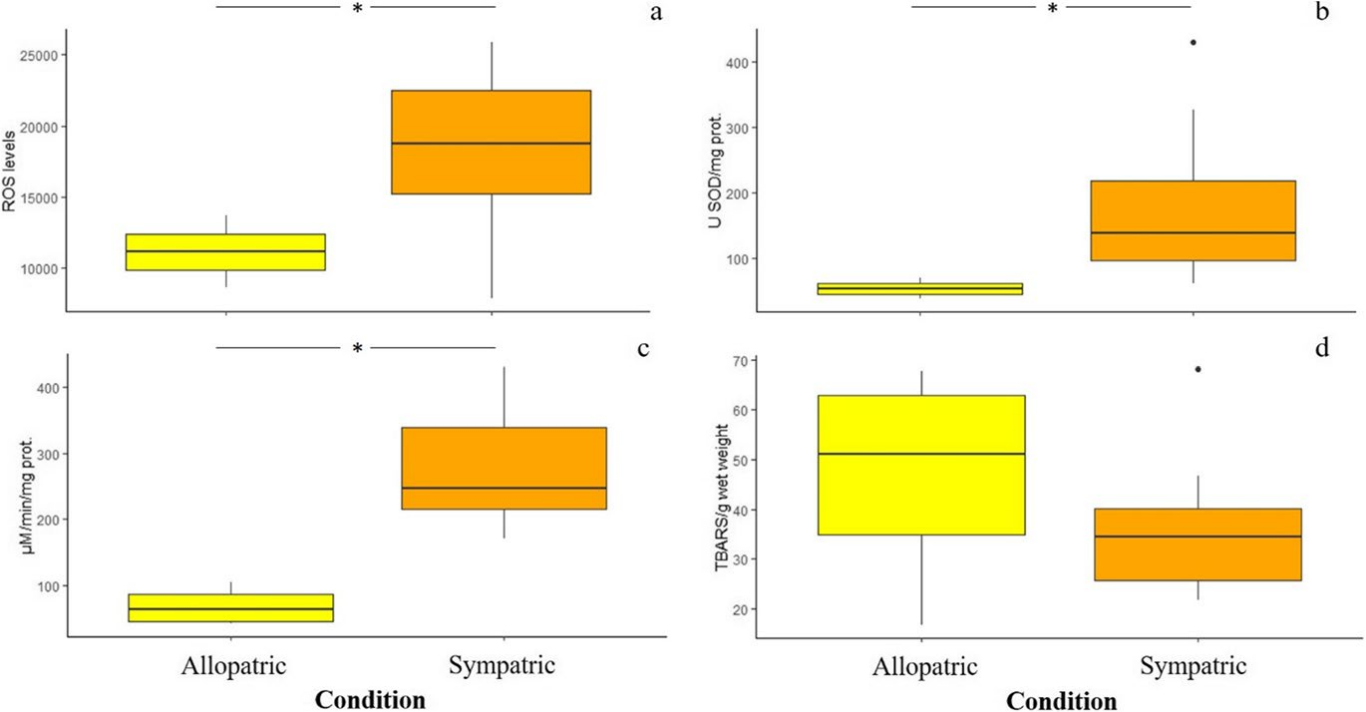
Boxplots of the effects due to interspecific competition on the levels of ROS (**a**), the activity of antioxidant enzymes (SOD and CAT, **b** and **c**), and levels of lipid peroxidation (**d**) in the liver of the brown trout. The asterisk indicates a statistically significant difference (*p < 0.05) between individuals of the brown trout under allopatric or sympatric conditions

The brown and the rainbow trout responded differently to interspecific competition. A modulation of the hepatic oxidative status in brown trout individuals experiencing sympatric conditions compared to conspecifics in allopatry was noted (Fig. 3). A significant increase in ROS levels (Fig. 3a) was noted in brown trout in sympatry with compared to conspecifics in allopatry (*F*_1,10_ = 4.334, *p* = 0.045). Accordingly, a significant activation of hepatic activity of the antioxidant enzymes SOD (*F*_1,14_ = 4.661, *p* = 0.040) and CAT (*F*_1,14_ = 23.213, *p* < 0.001) was noted in brown trout under sympatric conditions compared to those under allopatric ones (Fig. 3b and c). No significant effects of the experimental condition on GPx activity (*F*_1,11_ = 0.008, *p* = 0.978; data not shown) and oxidative damage in terms of lipid peroxidation levels (*F*_1,14_ = 1.459, *p* = 0.247; Fig. 3d) occurred.

**Fig. 4:**
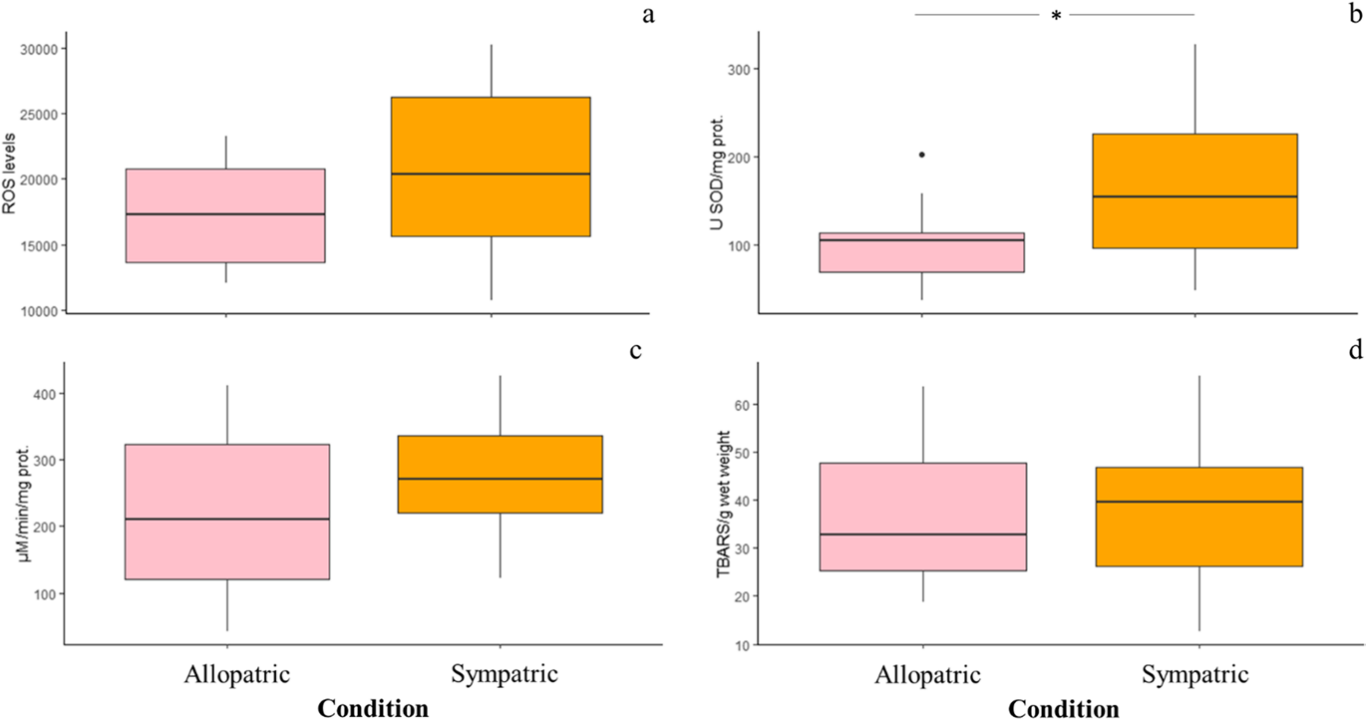
Boxplots of the effects due to interspecific competition on the levels of ROS (**a**), the activity of antioxidant enzymes (SOD and CAT, **b** and **c**), and levels of lipid peroxidation (**d**) in the liver of the rainbow trout. No statistically significant difference (*p* > 0.05) occurred between individuals of the rainbow trout under allopatric or sympatric conditions, with the exception of SOD activity (*p < 0.05)

No significant modulation of oxidative status, in terms of ROS levels (*F*_1,19_ = 1.812, *p* = 0.194), CAT (*F*_1,20_ = 1.503, *p* = 0.234), and GPx (*F*_1,18_ = 4.149, *p* = 0.056) activity, as well as oxidative damage such as lipid peroxidation (*F*_1,19_ = 0.022, *p* = 0.882), was noted in individuals of the rainbow trout experiencing interspecific competition (Fig. 4a–d). However, a significant increase in SOD activity (*F*_1,20_ = 5.040, *p* = 0.036) was observed in rainbow trout maintained in sympatric populations compared to allopatric ones.

Overall, no significant modulation of the oxidative status and oxidative damage occurred in the gills of individuals from both the species maintained under sympatric or allopatric conditions (see Supplementary Information, Table S1).

## Discussion

The results of the present study showed that the competitive interactions among parr of nonnative salmonid species, such as the rainbow and the brown trout, resulted in phenotypic consequences and physiological alterations in brown trout individuals only, while very slight effects occurred in rainbow trout individuals.

The 2-month-long sympatric interactions between brown and rainbow trout parr induced a significant reduction of the specific growth rate (SGR) in brown trout individuals compared to conspecifics under allopatric condition, while a slight non-significant reduction was noted for the rainbow trout (Fig. 2). Our findings suggested that competitive interactions between these two salmonid species can result in negative phenotypic consequences for the brown trout. This outcome was the opposite compared to that of a previous manipulative experiment performed on the same trout species (Hasegawa 2016). Indeed, such a study showed that 15-day-long sympatric interactions among young-of-the-year (YOY) individuals affected the SGR of the rainbow trout, but not of the brown trout. Specifically, a decrease in SGR, coupled with a reduction of stomach content, was noted in rainbow trout YOY maintained in sympatry with the brown trout, as well as in high-density allopatric populations. In contrast, no effects on SGR and stomach content occurred in brown trout YOY, under neither allopatric nor sympatric conditions, at low or high population density (Hasegawa 2016). The discrepancy in the species-specific outcomes of interspecific competition might be due to the huge differences in experimental conditions adopted by the studies. Indeed, the duration of the competitive interactions, the population density, and the life stage (i.e., the age) of individuals stocked in the experimental pools can affect the intensity and the outcomes of intra- and interspecific competitive interactions (Prati et al. 2021). Although we did not investigate the stomach content of trout, our results suggest that in sympatric populations parr of the rainbow trout can be considered better antagonistic competitors than brown trout. Brown trout could experience limited access to food resources, suffering a decrease in SGR. Food limitation might be likely due to interference competition among individuals of both the species and/or the consequent shortage of available foraging habitat rather than exploitative competition (Grant and Imre 2005; Imre et al. 2005; Hasegawa 2016). The rainbow trout might force the brown trout to use unsuitable foraging habitats, such as the corner of the experimental pools, or to feed on alternative low-value preys, limiting their food ingestion and energy intake for body growth (Yamamoto and Reinhardt 2003; Prati et al. 2021 and references therein). Alternatively, as in field experiment food resources could not be controlled, phenotypic and physiological (see below) effects of sympatric interactions might be mediated by changes in behavior, such as the burst of spontaneous aggressive behavior. As fish individual growth reflects the balance between energy intake and costs due to baseline metabolic activity (e.g., Deslauriers et al. 2017), it integrates the effects of biotic interactions and increased activity caused by a burst of agonistic behavior for foraging success (Pennock et al. 2022). However, although manipulative experiments represent a valuable approach to shed light on the outcomes of intra- and interspecific competition under natural field condition, they suffer some sacrifice of realism (e.g., Underwood 2012). Thus, the decrease in SGR of brown trout might be, at least partially, an experimental artifact, caused for instance by the reduced invertebrate preys entering into our field enclosures (e.g., Cooper et al. 1990; Pennock et al. 2022). All these hypotheses needs to be confirmed through observations of differences in stomach content, as well as aggressive and foraging behavior of individuals of both the species experiencing sympatric interactions. The decrease in the SGR observed in the brown trout (and limitedly in the rainbow trout) maintained under sympatric conditions might originate from a reduced food ingestion and/or spontaneous activity associated with agonistic behavior. These situations can cause enhanced metabolic rates and increased in oxygen uptake and usage (Li and Brocksen 1977; Barton and Iwama 1991; Laursen et al. 2013) at low population density (Laursen et al. 2013), leading to changes in antioxidant defenses and the onset of an oxidative stress condition. Indeed, fish antioxidant defenses can be affected by a series of intrinsic (e.g., systematic position, age, feeding behavior, food consumption, and diet) and extrinsic (e.g., contaminant exposure, seasonal and daily changes in dissolved oxygen and water temperature) factors (Martínez-Álvarez et al. 2005; Aras et al. 2009; Solé et al. 2009). An overall significant modulation of the hepatic oxidative status occurred in individuals of the brown trout experiencing sympatric conditions compared to conspecifics maintained in allopatry. In contrast, only a very slight modulation of the oxidative status (i.e., increase in SOD activity) was noted in rainbow trout individuals maintained in sympatry compared to conspecifics in allopatry (Fig. 4). A significant increase in hepatic ROS levels accompanied by the activation of antioxidant enzymes SOD and CAT was noted in brown trout interacting with rainbow trout compared to conspecifics maintained in allopatry (Fig. 3 a–c). The overall activation of SOD suggests that this enzyme tackled the overproduction of superoxide (O_2_^−^) radicals generated from competitive interactions among trout through their dismutation in molecular oxygen (O_2_) and hydrogen peroxide (H_2_O_2_), whose toxicity was counteracted by the activation of CAT (Chowdhury and Saikia 2020). From a physiological point of view, the enhancement of ROS levels could be due to the increase of physical activity and/or aggressive behavior due to interference competition, which can boost the oxygen metabolism at mitochondria level (Alessio and Goldfarb 1988). Alternatively, as observed on different fish species, food limitation and/or starvation can cause a ROS overproduction and the consequent activation of enzymatic antioxidant defenses. For instance, an increase in antioxidant enzyme activity (i.e., SOD and GPx) was noted in the liver of fasted gilthead seabream (*Sparus aurata*) individuals (Pascual et al. 2003), while an activation of SOD, CAT, GPx, and glutathione reductase (GR) was observed in the liver and the gills of brown trout during a prolonged starvation period (Bayir et al. 2011). Thus, the putative decrease in food ingestion might limit the intake of dietary non-enzymatic antioxidants, promoting the activation of antioxidant enzymes to counteract the toxicity of ROS and to prevent the onset of an oxidative stress condition. In fact, no significant increase of oxidative damage, in terms of lipid peroxidation levels (Fig. 3d), was noted in both the trout species, confirming the effectiveness of antioxidant enzyme machinery. Alterations in oxidative status occurred only in the liver of trout under sympatric conditions compared to conspecifics in allopatry, while no changes were observed in the gills (Table S1). Many studies performed on fish, including the rainbow and the brown trout (Otto and Moon 1996; Bayir et al. 2011; Parolini et al. 2019), showed that antioxidant responses vary in relation to the tissues (e.g., Pascual et al. 2003). Overall, the hepatic antioxidant capacity, including the enzyme defenses, is higher than in other organs (Perez-Campo et al. 1993; Otto and Moon 1996; Parolini et al. 2019) because of the high oxygen metabolism and consumption, which lead to a higher generation of ROS in the liver (Gomez et al. 2010). In contrast, competitive interactions did not altered the metabolism of gills neither in the rainbow trout nor in the brown trout, suggesting a low generation of ROS in gills that did not require the activation of antioxidant enzymes. These findings confirmed that the liver is the best organ to investigate the changes in the oxidative status of fish in response to different environmental stressors (Wilhelm-Filho et al. 1993), including competitive interactions.

## Conclusions

The present study demonstrated that sympatric interactions between parr of the rainbow and the brown trout resulted in negative consequences on the body growth of the brown trout only. These results could be probably due to a reduction in food availability and/or ingestion of low-value food, as well as behavioral changes related to the enhancement of physical activity or aggressive behavior because of interference in competition by the rainbow trout. Interspecific competition induced physiological alterations, such as the alteration of the oxidative status related to the activation of antioxidant enzymatic defenses, in individuals of the brow trout maintained in sympatry compared to conspecifics in allopatry. Such modulation could be due to the increase of physical activity during interference competition for food supply and/or to a limited intake of dietary non-enzymatic antioxidants, which promoted the activation of enzyme machinery to tackle ROS overproduction. However, further studies exploring the differences in stomach content, as well as changes in non-enzymatic antioxidants accumulated in trout tissues, should allow to verify our hypotheses and to explain the outcomes of interspecific interactions between the rainbow and the brown trout. In addition, considering that the outcomes of competitive interactions depend on the age of individuals, manipulative field studies investigating the interactions among individuals of different age classes at different population densities should be crucial to shed light on potential temporal or spatial variations in resource use, population, and individual niche width of trout species experiencing sympatric interactions. These findings should allow us to understand the population dynamics of these nonnative salmonid species and to identify the drivers leading to the establishment of a species rather than the other one in freshwater ecosystems.

### Supplementary Information

Below is the link to the electronic supplementary material.

Supplementary file1 (DOCX 16 KB)

## Data Availability

Data of the experiment will be available upon request.
