# Supplementary material for: Interspecific competitive interactions affect body size and oxidative status of two nonnative salmonid species
Source: Fish Physiol Biochem. 2024 Jan 19;50(2):721–32. doi: 10.1007/s10695-024-01301-0 (PMC11021341; doi:10.1007/s10695-024-01301-0)
Supplement: Supplementary file 1 — Supplementary file1 (DOCX 16 KB) [file 10695_2024_1301_MOESM1_ESM.docx]

**Supplementary information**

**Table S1:** effects of experimental condition (allopatry vs sympatry) on oxidative stress parameters (i.e., ROS level, activity of the antioxidant enzymes SOD, CAT and GPx, as well as lipid peroxidation) measured in the gills of the brown and rainbow trout.

|  | **F** | **df** | **p** |
| --- | --- | --- | --- |
| ***Brown trout*** |  |  |  |
| **ROS** |  |  |  |
| Condition | 1.551 | 1, 13 | 0.234 |
|  |  |  |  |
| **SOD** |  |  |  |
| Condition | 1.859 | 1, 14 | 0.192 |
|  |  |  |  |
| **CAT** |  |  |  |
| Condition | 2.145 | 1, 14 | 0.165 |
|  |  |  |  |
| **GPx** |  |  |  |
| Condition | 1.581 | 1, 14 | 0.229 |
|  |  |  |  |
| **Lipid peroxidation** |  |  |  |
| Condition | 0.445 | 1, 12 | 0.518 |
|  |  |  |  |
| ***Rainbow trout*** |  |  |  |
| **ROS** |  |  |  |
| Condition | 2.471 | 1, 18 | 0.128 |
|  |  |  |  |
| **SOD** |  |  |  |
| Condition | 0.793 | 1, 19 | 0.348 |
|  |  |  |  |
| **CAT** |  |  |  |
| Condition | 2.074 | 1, 19 | 0.166 |
|  |  |  |  |
| **GPx** |  |  |  |
| Condition | 2.154 | 1, 18 | 0.159 |
|  |  |  |  |
| **Lipid peroxidation** |  |  |  |
| Condition | 0.009 | 1, 16 | 0.923 |
